# Challenging the foundations of the clinical model of foot function: further evidence that the root model assessments fail to appropriately classify foot function

**DOI:** 10.1186/s13047-017-0189-2

**Published:** 2017-02-03

**Authors:** Hannah L. Jarvis, Christopher J. Nester, Peter D. Bowden, Richard K. Jones

**Affiliations:** 1 School of Health Sciences, University of Salford, Salford, UK; 2 Department of Exercise and Sport Science, Manchester Metropolitan University, Crewe Campus, Crewe, UK

**Keywords:** Static, Dynamic, Assessment, Neutral

## Abstract

**Background:**

The Root model of normal and abnormal foot function remains the basis for clinical foot orthotic practice globally. Our aim was to investigate the relationship between foot deformities and kinematic compensations that are the foundations of the model.

**Methods:**

A convenience sample of 140 were screened and 100 symptom free participants aged 18–45 years were invited to participate. The static biomechanical assessment described by the Root model was used to identify five foot deformities. A 6 segment foot model was used to measure foot kinematics during gait. Statistical tests compared foot kinematics between feet with and without foot deformities and correlated the degree of deformity with any compensatory motions.

**Results:**

None of the deformities proposed by the Root model were associated with distinct differences in foot kinematics during gait when compared to those without deformities or each other. Static and dynamic parameters were not correlated.

**Conclusions:**

Taken as part of a wider body of evidence, the results of this study have profound implications for clinical foot health practice. We believe that the assessment protocol advocated by the Root model is no longer a suitable basis for professional practice. We recommend that clinicians stop using sub-talar neutral position during clinical assessments and stop assessing the non-weight bearing range of ankle dorsiflexion, first ray position and forefoot alignments and movement as a means of defining the associated foot deformities. The results question the relevance of the Root assessments in the prescription of foot orthoses.

## Background

The first definitive protocol for clinical biomechanical assessment of the foot was developed by Root et al. [1, 2] which is often referred to as the “Root model” of foot function. The core concepts continue to be prominent in popular texts [3–6], debates, conferences [7–10], practice [11–17], undergraduate podiatry syllabus across the United Kingdom [Nester, personal communication, December 2016] and are highly prevalent in grey literature and online resources. These include using static assessment of the foot to infer dynamic foot kinematics [15], defining structural deformities between foot segments and advocating their correction [4, 5], and using foot shape when the sub talar joint is in a ‘neutral positon’ as a basis for orthotic design[4, 11, 16]. The Root model was based on the premise that in a “normal” foot the bones and joints demonstrate specific biomechanical alignments and ranges of motion and that these can be measured in a static (non-weight bearing or standing) biomechanical assessment. Abnormal alignments or movement range could also be identified through this static assessment of the foot and were classified as ‘deformities.’ Different deformities were supposedly associated with specific and ‘pathological’ compensatory movements during walking, and were assumed to be the cause of a wide range of clinical symptoms. Thus, a foot can be classified as abnormal or normal based on what is observed clinically and this can predict what occurs dynamically during gait. Furthermore, the degree of abnormal alignment or movement would correlate with the degree of compensation.

Whilst easy to conduct within routine clinical practice there is now compelling evidence of poor intra and inter-clinician reliability of the protocols Root et al. [1, 2] proposed to identify these abnormal alignments and movements[13, 18–23]. Despite this, clinicians persist in the use of these examinations. However, few studies have tested the validity of the classifications, i.e. (1) whether feet classified with different structural abnormalities of the rear, mid and forefoot exhibit different kinematics during gait, and (2) whether the degree of abnormality correlates with the degree of abnormal compensatory movement during gait.

Whilst there were a range of assessments advocated by Root et al. [1, 2] there are five that are particularly central to their model of clinical foot function and remain relevant to current practice [13]. Examination of the frontal plane position of the subtalar joint in so called *neutral* and *relaxed* calcaneal stance positions (NCSP and RCSP respectively) is arguably the cornerstone of the Root et al. [1, 2] assessment protocol. Use of this assessment assumes that in the normal foot the heel will be vertical when standing in NCSP and that the sub talar joint will pass through its neutral position in mid stance. Passing through the neutral position is thus synonymous with normal foot function. If the subtalar joint is inverted in NCSP the foot is classified as abnormal and as having a ‘rearfoot varus’ deformity. As compensation, Root et al. [1, 2] proposed that the subtalar joint would evert during mid-stance exactly the same number of degrees it is inverted in NCSP. Therefore, there will be a correlation between the degree of rearfoot varus (the deformity) and the degree of mid stance eversion (the compensation). McPoil and Cornwall [24] and Pierrynowski and Smith [25] report that in pain free, i.e. normal healthy, participants the subtalar joint is not in a vertical position in NCSP, nor does the heel evert the same number of degrees it was inverted in NCSP. Also, critically, they reported that the heel does not pass through the sub talar neutral position during stance. However, in these studies the techniques used to place the foot into NCSP for the static assessment were not precisely as Root et al. [1] described them. Further investigation of these fundamental aspects of the Root et al. [1, 2] protocol is therefore warranted.

Root et al. [1, 2] also proposed that a prerequisite for a normal rearfoot was the ability of the ankle to dorsiflex 10° during gait and that the availability of this 10° could be tested in a static examination. In feet classified as unable to dorsiflex to 10°, described as an ‘ankle equinus’ deformity, Root et al. [2] proposed that the subtalar joint will undergo more pronation to compensate for the limited range of ankle dorsiflexion. However, recent publications [26–29] indicate that most symptom free ankle joints do not possess the ability to dorsiflex to 10°. Furthermore, the range of subtalar joint pronation is similar between feet with and without ankle equinus [30].

In the forefoot, the Root et al. [1] protocol advocates assessment of the sagittal plane position of the first ray (first metatarsal and medial cuneiform) and the frontal plane alignment of the forefoot relative to the rearfoot. For the former, the first ray can be classified as either abnormally dorsiflexed or plantarflexed, with plantarflexion affecting rearfoot eversion, and dorsiflexion limiting 1^st^ metatarsophalangeal joint (MPJ) dorsiflexion. The latter is a classification of varus or valgus alignment of the forefoot relate to the plantar surface of the calcaneus. Greater varus or valgus malalignment of the forefoot is thought to correlate with greater degrees of compensatory rearfoot eversion. Thus, in both cases, feet classified with the deformity are thought to exhibit different kinematics compared to a normal foot. However, the validity of these hypotheses has not been tested comprehensively [31].

Also related to the forefoot, Root et al. [1, 2] specified that in the normal foot the first metatarsophalangeal joint must be able to dorsiflex 65° during late stance and that the availability of this motion during gait could be tested in a static assessment. Feet classified as having less than 65° in a static assessment would demonstrate <65° during propulsion, and the greater the loss of dorsiflexion the greater the compensatory rearfoot pronation required. However, Halstead and Redmond [32], and others [33–35], report that most symptom free feet are unable to achieve 65° of dorsiflexion, questioning its validity as an means of identifying normal and abnormal foot function.

The Root et al. [1, 2] protocol for static assessment of the foot thus assumes a relationship exists between classification of feet with abnormal alignment (rearfoot varus, forefoot varus/valgus, 1^st^ ray position) or range of motion (ankle equinus, 1^st^ MPJ dorsiflexion) and foot kinematics during gait. Specifically, feet classified with these structural deformities will exhibit different foot kinematics during gait compared to those without, because the deformities cause specific compensations to occur. Furthermore, that the extent of the abnormal alignment/movement is associated with the degree of compensatory motion. Thus, a correlation should exist between the scale of the abnormality and the scale of the compensatory motion that results. The definition of these abnormalities relies upon the validity of the sub talar neutral position as a suitable reference point for normal foot kinematics, specifically that it is a position adopted by normal feet during gait. The aim of this study therefore, is to: (1) determine whether foot kinematics during gait are different between feet with and without the five key structural deformities described by Root et al., [1, 2] (2) to investigate any correlation between the degree of structural deformity and degree of compensatory foot kinematics during gait, and (3) to test whether symptom free feet utilise the subtalar neutral position during gait.

## Method

### Recruitment

Ethical approval was granted by the University of Salford ethics committee and all participants provided written consent. Through advertising, introductory presentations and workshops a convenience sample of 140 asymptomatic and self-reported healthy individuals aged 18–45 were recruited from a University student and staff population. Medical history (including current and prior medication), vascular assessment (palpation of foot pulses), neurological assessment (vibration perception using 128Hz fork, light touch perception using 10 g monofilaments), and calculation of BMI were undertaken on both feet. However as recommended by Menz [36] only the left was used for static assessment or measurement of foot kinematics. Participants were excluded if they had history of musculoskeletal disease such as rheumatological conditions (e.g. rheumatoid/psoriatic or osteo-arthritis), foot or lower limb pain in last 6 months, had BMI less than 16 or greater than 30, had worn foot orthoses previously, and presented with any sign of compromised vascular or neurological status. Participants were excluded if either foot displayed hallux-abducto valgus indicated as lateral deviation of the hallux and medial metatarsal prominence. Screening against these criteria identified 100 participants and they were asked to self report their physical levels on a 5 point scale (1 = not active at all, 5 = active 5 times a week). All data were collected from each participant during a single visit.

### Static assessment

The static examinations consisted of NCSP, RCSP, range of dorsiflexion at the ankle joint, range of dorsiflexion at the 1^st^ MPJ, position and range of motion of the first ray, and frontal plane position of the forefoot to rearfoot as described in Jarvis et al. [13]. This identified: the presence and any extent of rearfoot varus, ankle equinus, forefoot varus/valgus/normal, 1^st^ ray dorsiflexed/normal/plantarflexed positon, and maximum range of 1^st^ MPJ dorsiflexion. Angular measures for NCSP were made using a Digital Biometer (Langer Group, USA), a two axis flexible goniometer (Biometrics Ltd, Motion Lab Systems, LA, USA) for the range of ankle dorsiflexion and a finger goniometer (Health and Care, London, GB) for the range of dorsiflexion at the 1^st^ MPJ. The frontal plane forefoot/rearfoot relationship was classified via visual inspection as per Root et al. [1, 2] protocol. All assessment were performed by one assessor with >30 years’ experience.

To allow our sample to be compared to the feet investigated in other literature, the Foot Posture Index (FPI) was also recorded [37].

### Kinematic data

A 6 segment model (leg, calcaneus, midfoot (navicular and cuboid), lateral forefoot (fourth and fifth metatarsals), medial forefoot (first metatarsal) and hallux was used to characterise foot kinematics as described in Nester et al. [38]. Rigid plastic plates were heat molded to plaster casts of size 4 and 6 female feet, and sizes 9 and 12 male feet to enable improved fitting for different foot sizes. Each plate had three or four (leg only) 7 mm markers attached as described in Nester et al. [38]. Placement of plates on appropriate underlying bones was assisted through manual palpation and manipulation of adjacent joints (e.g. flexing/extending the fifth metatarsal to establish the location of the cuboid-metatarsal joint).

Kinematic data were collected using 12 100Hz cameras (Qualisys, Sweden). Force plate data (AMTI, 1500Hz) was collected to determine the start and end of stance/swing. Participants walked at their own self-selected speed and eight walks were recorded. A standing reference trial was collected to define 0° in the kinematic data. For the standing trial anatomical markers were placed on medial and lateral knee joint margins and the medial and lateral malleoli. A further static standing trial was recorded during which the rearfoot was placed into sub talar neutral.

Kinematic data were processed in Visual3D (C Motion, Rochelle, USA) and low pass filtered (6Hz, Butterworth). For each of the five foot segments and the leg a local co-ordinate system (LCS) was defined using the reflective markers. The vertical (z) axis of the leg LCS was a line joining the midpoint of the malleoli distally, and midpoint of the medial and lateral knee margins proximally. The anterior/posterior axis (y) was determined by the unit vector perpendicular to the frontal plane that was a least squares plane through the z axis and the four anatomical markers on the knee and malleolus. The medial/lateral (x) axis was perpendicular to z and y. The foot segment LCS axes were all set parallel to those of the leg LCS during the standing reference trial.

Angular motion was calculated for five inter-segment combinations that were assumed to have six degrees of freedom: Rearfoot (calcaneus-tibia), midfoot-calcaneus, medial forefoot-midfoot, lateral forefoot-midfoot and hallux-medial forefoot (1^st^ MPJ) (Cardan sequence x-y-z). The mean of eight walking trials was derived.

Matlab (R2014a, Mathworks) was used to extract data variables. Variables were chosen according to the compensatory movements that Root et al. [2] proposed would occur for each of the five structural abnormalities (Table 1). For continuity with prior use [38] of the data set and to maintain independence of data, left foot data was used.

**Table 1:** Details the static variables used to define the rearfoot, ankle, first ray, forefoot and hallux deformities investigated, the foot kinematic variables used to compare feet with and without the deformities, and the foot kinematic variables used in the assessment of any correlation between the degree of deformity and degree of compensatory foot motion

Deformity (and comparator group)	Static variable used to define deformity	Dynamic variables used in *group comparisons*	Dynamic variables used in *correlation analysis*
Rearfoot varus Rearfoot valgus	Frontal plane angle of the calcaneus relative to the floor^b^	n/a too few data	Peak rearfoot eversion angle during mid-stance
Ankle equinus (vs no ankle equinus)	Range of dorsiflexion at the ankle joint^b^	Peak dorsiflexion angle of the rearfoot during mid-stance Peak eversion angle of the rearfoot during mid-stance	Sagittal plane angle of the rearfoot at heel lift Peak rearfoot dorsiflexion angle of the during mid-stance Range of sagittal plane rearfoot motion during mid-stance
Plantarflexed first ray (vs no first ray deformity)	Sagittal plane position of first ray^a^	Peak eversion angle of the rearfoot during mid-stance	n/a^a^
Dorsiflexed first ray (vs no first ray deformity)	Sagittal plane position of first ray^a^	Peak dorsiflexion angle of the hallux-medial forefoot during propulsion	n/a^a^
Forefoot varus Forefoot valgus (vs no forefoot to rearfoot deformity)	Frontal plane position of the forefoot relative to rearfoot^a^	Peak eversion angle of the rearfoot during mid-stance	n/a^a^
<65° Hallux dorsiflexion (vs >65° hallux dorsiflexion)	Maximum angle of hallux dorsiflexion^b^	Peak sagittal plane angle of the hallux-medial forefoot during propulsion Peak eversion angle of the rearfoot during propulsion	Peak sagittal plane angle of the hallux-medial forefoot during propulsion Peak rearfoot eversion angle during propulsion

^a^The static assessment of these deformities relied on a binary classification (i.e. present/absent) and is therefore not suitable for correlation analysis. ^b^used in correlation analysis

### Statistical analysis

Statistical analysis was conducted using IBM SPSS (Statistical Package for the Social Sciences) Version 20 (IBM Corporation, New York, USA). All data were checked using the Kolmogorov Smirnov test and parametric/non parametric test used accordingly. For a two group comparisons (ankle equinus and less than 65° 1^st^ MPJ dorsiflexion) an independent t-test or Mann–Whitney test was used. For the three group comparisons (first ray, forefoot to rearfoot relationship) a one-way ANOVA with post-hoc analysis using Least Significant Difference was used, or a Kruskall-Wallis test with post-hoc analysis was employed, using individual Mann–Whitney tests with Bonferroni correction. To investigate relationships between data (Table 1) a Pearson correlation was used for parametric data and a spearman correlation for non-parametric data.

To investigate whether the rearfoot of each participant passed through sub talar neutral during gait, the frontal plane rearfoot angle when standing in sub talar neutral was compared to the frontal plane rearfoot position during gait. Where the two angles coincided, the time during gait when this occurred was derived. Where angles did not coincide the minimum difference between the two was derived.

## Results

**Fig. 1:**
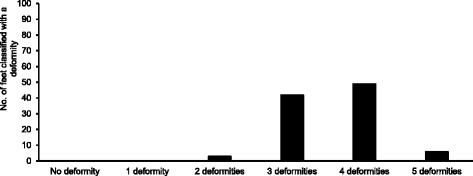
Presents the number of feet classified with 0,1,2,3,4 or 5 structural deformities of the foot

**Fig. 2:**
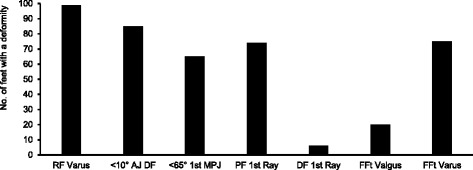
Presents the number of feet classified with a specific type of structural deformity: RFt varus = rearfoot varus, <10 AJ DF = Ankle equinus, <65° 1^st^ MPJ DF = range of hallux dorsiflexion <65°, PF 1^st^ ray = plantarfexed first ray, DF 1^st^ ray = dorsiflexed first ray, FFt valgus = forefoot valgus, Fft varus = forefoot varus

**Fig. 3:**
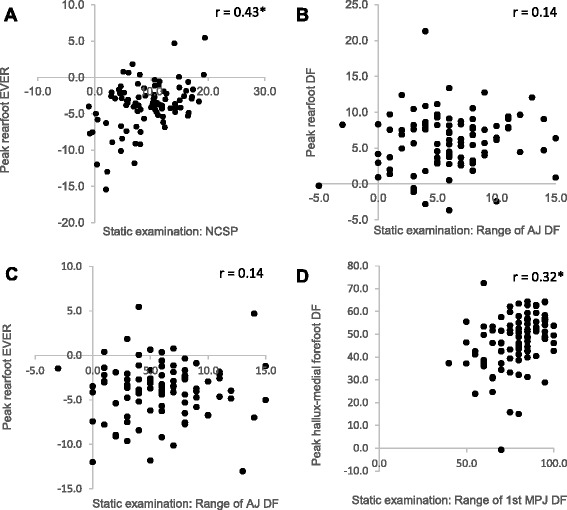
Presents correlation matrices for NCSP and peak rearfoot eversion (**a**), range of dorsiflexion at the ankle joint and peak rearfoot dorsiflexion (**b**), range of dorsiflexion at the ankle joint and peak rearfoot eversion (**c**), range of first metatarsophalangeal dorsiflexion and peak hallux-medial forefoot dorsiflexion (**d**). NCSP = Neutral calcaneal stance position, DF = Dorsiflexion, EVER = eversion, MPJ = metatarsophalangeal. *indicate significant correlation (*p* = <0.05)

**Fig. 4:**
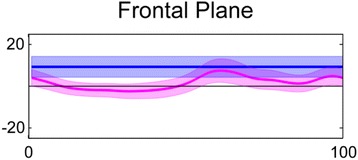
Presents frontal plane kinematics of the rearfoot in feet classified with a rearfoot varus

Data describing participants are detailed in Table 2. All feet were classified with at least two structural deformities of the foot (Figs. 1 and 2). In terms of the general patterns in the foot kinematics there very few differences between feet classified with or without a deformity (Fig. 4). There was only one statistically significant difference in foot kinematics between the feet with and without a structural deformity (related to feet with less than 65° 1^st^ MPJ dorsiflexion). For all five of the Root et al. [1, 2] static assessments, there was no strong nor moderate correlations between any of the static and dynamic parameters (Fig. 3). The strongest significant correlation was *r* = 0.43 (*p* < 0.001) for between NCSP and peak rearfoot eversion (Fig. 3a). Other correlations were all *r* = <0.32.

**Table 2:** Presents participant characteristics. Mean (95% CI)

	Age (yrs)	Height (cm)	Mass (kg)	Activity level*	FPI Pronated	FPI Neutral	FPI Supinated
All participants							
(Male and Female)	31.7 (28.5- 35.1)	168.3 (166.7- 169.9)	71.8 (69.0- 74.6)	3.2 (2.9- 3.4)	7.7 (7.1- 8.2)	2.6 (2.1- 3.1)	−2.4 (−1.6- -3.2)
*n* = 100					32%	56%	12%
Female participants *n* = 71	31.5 (27.7- 35.3)	164.8 (163.5- 166.1)	68.0 (64.9- 71.2)	3.1 (2.9- 3.3)	7.6 (8.3- -6.9)	2.6 (2.0- 3.2)	−2.1 (−2.9- -1.3)
Male participants *n* = 29	32.3 (25.7- 38.9)	176.9 (174.2- 179.5)	81.0 (76.5- 85.6)	3.4 (2.9- 3.8)	7.7 (6.7- 8.8)	2.3 (1.4- 3.1)	−2.5 (−2.0- -3.0)

**Fig. 5:**
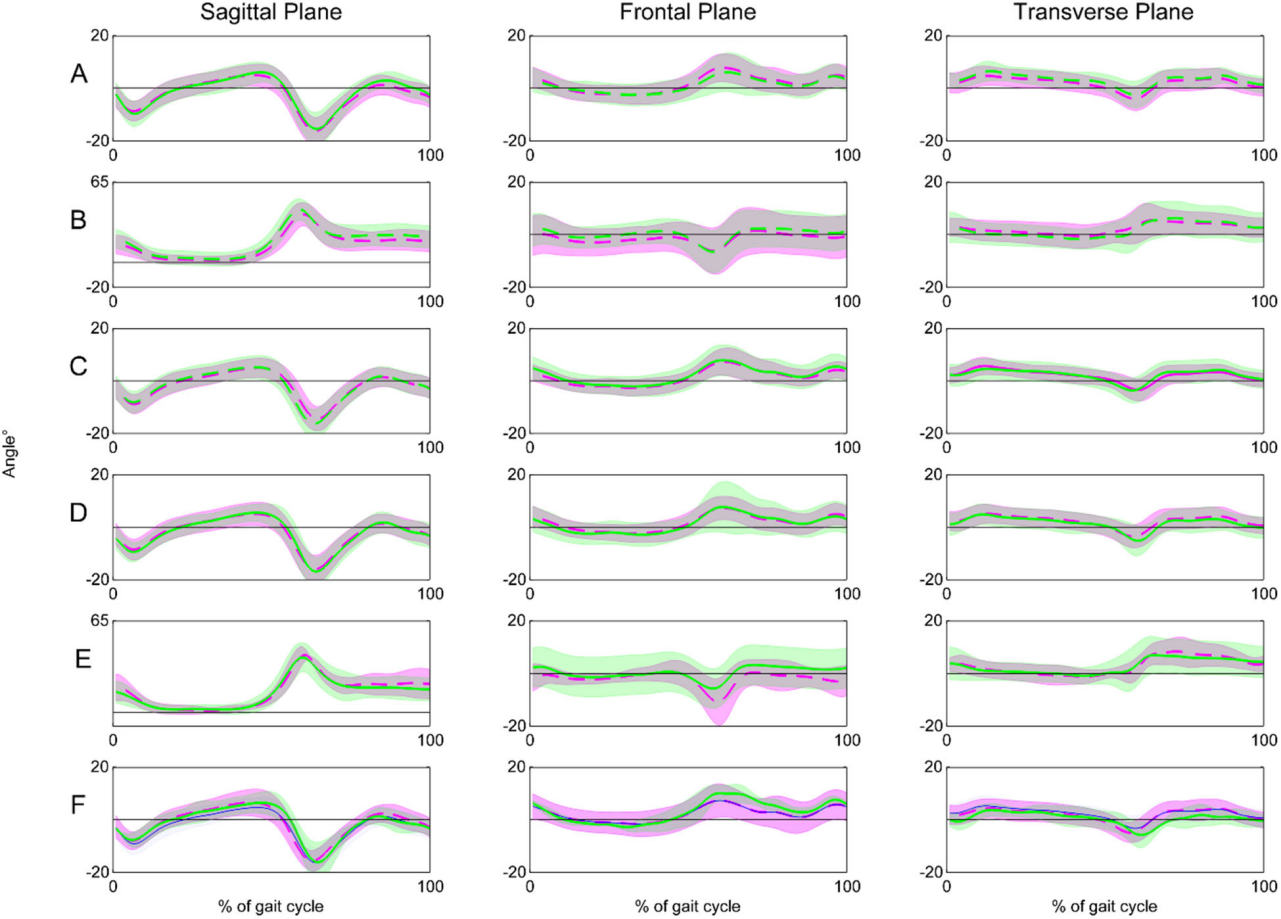
Presents foot kinematics in feet with and without each of the rearfoot, ankle, first ray, forefoot and hallux deformities investigated (refer to Table 1 for definition of dynamic variables used in group comparisons). *Pink* or *blue* refers to the deformity group in all cases, *green* = no deformity. **a** = rearfoot motion in ankle equinus *vs.* no equinus deformity, **b** = hallux-medial forefoot motion in <65° Hallux dorsiflexion deformity *vs.* no hallux deformity (i.e. >65° dorsiflexion), **c** = rearfoot motion in <65° Hallux dorsiflexion deformity *vs.* no hallux deformity (i.e. >65° dorsiflexion), **d** = rearfoot motion in plantarflexed 1^st^ Ray deformity *vs.* no 1^st^ ray deformity. **e** = hallux-medial forefoot motion in dorsiflexed 1^st^ Ray *vs.* no 1^st^ ray deformity, **f** = rearfoot motion in forefoot varus vs. forefoot valgus vs. no forefoot deformity

The calcaneus was inverted in NCSP in 97% of feet and thus there were too few feet without rearfoot varus for group comparisons. Mean rearfoot varus angle was 9.2° (8.2-10.3). During mid-stance, the rearfoot was not everted at heel lift (−0.7° (−1.5- 0.1) the same angle it was inverted in NCSP for any participant (Fig. 4). The rearfoot passed through or was closet to NCSP at 56.7% (55.6–57.8%) of the gait cycle, i.e. during late propulsion (Fig. 4). In all 39% of feet passed through sub talar neutral during stance.

The majority of feet (*n* = 85, 87%) were classified with ankle equinus (mean static dorsiflexion of 4.8° (4.2- 5.4) vs. 11.9° (10.9- 12.9)). Maximum dorsiflexion during stance was not statistically significantly different between feet with and without ankle equinus (5.6° (4.8- 6.5) compared to 6.6° (4.6- 8.6), *p* = 0.195). Similarly, the peak angle of rearfoot eversion during mid-stance was not statistically significantly different between those with (−3.9° (−4.7- -3.3)) and without ankle equinus (−4.1° (−6.2- -1.9)) (*p* = 0.363) (Fig. 5).

All (100%) feet were classified with at least one forefoot deformity, with either a forefoot varus or valgus, and/or a first ray or first metatarsophalangeal joint deformity. Forefoot varus were identified in 76% of feet, valgus in 20% and only 4% had no frontal plane forefoot/rearfoot relationship deformity. Neither the peak nor the range of rearfoot eversion was different between the three categories: forefoot varus −2.7° (−4.8- -0.5), forefoot valgus −4.1° (−4.9- -3.4)), normal (−3.2° (−4.9- -1.5)) (*p* = >0.239) (Fig. 5).

For the first ray, 74% were classified as plantarflexed, 7% dorsiflexed and 19% had no first ray deformity. Peak rearfoot eversion was not statistically different between feet classified with a plantarflexed (−6.5° (−7.7- -5.3)), or normal first ray (−4.8° (−9.7- -0.03)) (*p* = 0.206). Peak hallux-medial forefoot dorsiflexion of feet classified with a dorsiflexed first ray (42.5° (37.9- 47.1)) was not different to feet classified with no forefoot deformity (41.7° (36.3- 47.2) (*p* = 0.435) (Fig. 5).

Most feet (91%) were classified with greater than 65° of 1^st^ MPJ dorsiflexion. Feet classified with more than 65° dorsiflexion demonstrated significantly more dorsiflexion during propulsion (45.3° (43.3- 47.3) versus 39.3° (32.2- 35.2), *p* = 0.02)). Peak rearfoot eversion was not statistically significantly different between groups (less than 65° = −4.7° (−5.7- -3.7), greater than 65° = −3.7° (−4.5- -2.9) (*p* = 0.09)) (Fig. 5).

## Discussion

The results from this study, which is one of the largest to date and comprehensive in its investigation of the Root et al. [1, 2] protocol is in agreement with others [24, 25]. Together with the wider body of evidence, the results indicate that the assessments in the Root et al. protocol define foot deformities that have no relationship with foot kinematics during gait. This undermines their validity as the basis for identifying normal and abnormal foot function and as the basis for foot orthotic prescription.

None of the deformities proposed by Root et al. [1, 2] were associated with distinct differences in foot kinematics during gait, and static and dynamic parameters were not correlated. Like others [24, 25] and contrary to Root et al., [1, 2] our data indicate that if the subtalar joint is inverted in NCSP this bears no relationship to rearfoot kinematics during mid stance. This questions the perceived importance and continued clinical use of “subtalar joint neutral” to both define deformities and for capture of foot shape as part of foot orthosis prescription. For ankle equinus too, both Turner et al. [39] and McPoil and Cornwall [30] have previously failed to identify differences between feet with and without 10° of ankle dorsiflexion.

In most feet, the 1^st^ MPJ first could dorsiflex to 65° in the static assessment, but, consistent with other studies, the range of dorsiflexion used during propulsion was much less [32, 35, 40]. Similarly, Van Gheluwe [40] reported that in feet classified as having greater than 70° the first metatarsophalangeal joint dorsiflexed significantly more during propulsion than feet with less than 70°. However, the correlation between static and dynamic measures of dorsiflexion reported here is weak (*r* = 0.38), though slightly lower than in Van Gheluwe et al. [40] (*r* = 0.45). One weakness in the current investigation is the fact that the less than 65° group comprised only 9 ft. However, as with other assessments, this high incidence in a symptom free population undermines the very notion that less than 65° during walking is ‘abnormal’.

Indeed, this study concurs with others that the “abnormalities” proposed by Root et al. [1, 2] are common in symptom free populations. For all of the deformities described by Root et al. [1, 2] we found very high numbers in our sample as all feet were classified with at least two deformities, despite being symptom free (Figs. 1 and 2). This becomes a limitation of this study in that small groups are not suitable for statistical analysis, nor are groups of significantly different sizes. However, the high prevalence is in itself an important outcome of our work. The high number of cases in a symptom free group is good evidence that the deformities are in fact not deformities nor abnormalities at all. Indeed, other literature has identified these ‘deformities’ in symptom free populations. In Buchanan and Davis [41] 92% of 51 asymptomatic individuals were classified with a forefoot varus and similarly in Garbolosa et al. [42] 86.6% of 240 ft had a forefoot varus. Taken within the context of wider literature, the evidence presented here that large numbers of symptom free feet exhibit the so-called ‘structural deformities,’ and that these deformities are not associated with differences in foot kinematics, leads us to believe that the deformities are normal and irrelevant variations in foot alignment. This conclusion is further supported by evidence that the position into which the foot is placed to ‘diagnose’ these deformities (sub talar neutral) has been shown to be largely irrelevant for symptom free foot function. The Root et al. [1, 2] ideology assumes that feet presenting with these so called ’deformities’ will be symptomatic, that their function is ’abnormal’ and requires correction. We find no evidence of this in our data and the wider literature. The classifications proposed by Root et al. [1, 2] therefore appear to be invalid as determinants of foot function during gait and, assuming foot kinematics relate to the risk of injury, predictors of clinical injury.

It is important to understand why static measures are so poorly related to dynamic foot kinematics, which is an outcome of this, but also other literature [12, 17, 24, 30]. The static assessments are undertaken non-weight bearing or during standing, and the foot is manually positioned or moved by the clinician. The forces applied to the foot structures in a static examination bear little resemblance to those during gait in terms of magnitude nor direction. Furthermore, internal forces, especially from muscles, are largely absent except for some passive forces at extremes of joint position (e.g. ankle equinus). It follows, therefore, that the kinematics that result from the forces applied to the foot will differ from those during gait.

As part of a wider body of evidence the results of this study have profound implications for clinical foot health practice. We recommend that clinicians stop using the Root et al. [1] biomechanical examination protocol. It bears no or an uncertain relation to the position that healthy feet adopt during gait. Furthermore, the deformities defined when the foot is placed in the neutral positon do not appropriately classify differences in foot kinematics during gait. Assessment of the range of ankle dorsiflexion, first ray and forefoot alignments and movement seem erroneous since they too bear no relation to foot kinematics during gait. Based on the results here and the related literature over the last twenty years, we believe that the assessment protocol advocated by Root et al. [1] is no longer a suitable basis for professional practice.

Rather than focusing on identification of structural abnormalities that rely on unreliable subjective assessments, [13, 20, 23] greater emphasis should be placed on explaining the mechanical basis of symptoms and assessment of foot behaviour during weight bearing tasks that relate to symptoms. This could involve the conclusion that foot biomechanics are not implicated in the cause of some patient symptoms, a further point of difference from Root et al. [1, 2]. It follows that orthotic prescriptions might target changing stresses in specific (painful or at risk) structures rather than achievement of seemingly erroneous skeletal alignments. This would encourage a focus on assessing each patient in the context of their symptoms and personal and clinical context rather than comparing their feet to an unproven hypothetical model of an ‘ideal foot’. It would also allow definitions of “normal” to be created on a patient by patient basis, whereas Root et al. [1, 2] suggests we use orthoses to make foot movement the same or very similar in all patients. Finally, if the deformities described by Root et al. [1, 2] are the basis for prescribing some foot orthoses, then evidence that the so called deformities have no functional relevance, is perhaps evidence that foot orthoses should not be used in the absence of symptoms and simply to “correct” deformities. We believe this study, taken into context with the work of others, should signal the end of the clinical, educational and research use of Root et al. [1, 2] description of foot function and use of sub talar joint neutral position. The important innovation led by Root et al. [1, 2] was completed in the 1960’s and shortly thereafter, and they developed a theory in the absence of measurement approaches that could support systematic and objective investigation of their hypotheses, and how these could be related to symptoms experienced by their patients. In the intervening 40 or so years our ability to measure foot biomechanics has greatly improved, and the data describing foot biomechanics grown exponentially. We believe this and related works are an important milestone, building on but moving past the work of Root et al. [1, 2].

### Limitations

There are several limitations to this study. The difficulties of measuring foot movement with skin mounted markers are well documented [28, 38], although the Root et al. [1] assessment protocol also involved skin based markers to determine subtalar joint positon and movement, albeit statically, and these are subject to their own errors [23]. Like others before, this investigation used the movement of the calcaneus relative to the leg to represent movement of the subtalar joint. Without invasive methods it is not possible to measure the movement of individual bones and thus impossible to measure the actual sub talar joint, whose kinematics Root et al. [1, 2] refer to extensively. However, Root et al. [1, 2] also made many assumptions that lessen the impact of this issue. They often used the position of the heel relative to the floor to describe sub talar position, because they assumed the leg was vertical. Arguably, therefore, our approach is more faithful to the anatomical hypotheses under investigation and therefore has greater anatomical validity.

Prior research has consistently highlighted the variability between clinicians in the performance of the assessments we used to define the various foot deformities [13, 18–21, 23, 43]. Our reliance on one assessor to define the foot deformities therefore reduces the external validity of our study. However, controlling for known sources of variability in data is a prerequisite for quality research. If we had allowed more than one clinician to define the deformities the research outcomes might have been due to our inability to consistently define the deformities being investigated, and consistency is known to be better within one assessor than between several assessors [22]. Indeed, the fact that the assessments vary so much is simply a further problem with the Root model rather than an issue in our research. Indeedthe presence/absence of the deformities concerned can be dependent upon the clinician a patient sees rather than that actual arrangement of foot structures [13]. The high prevalence of some deformities likewise could be a result of bias in the assessor. However, given the poor reliability of the measures, this is again an issue with the reliability of the Root model rather than our research design. Involving more assessors to account for any assumed bias would have compromised our ability to identify the deformities as the independent variables in our research design.

There is a greater percentage of women than men recruited for this study which reflects our sampling of the local student population. Our focus was to investigate Root et al. [1, 2] model of foot function and no reference is made to gender specific deformities or compensations, rather there is a singular model of foot function that is valid independent of gender. As such the sample we used is a valid basis for testing the Root model [1, 2].

Whilst not strictly a limitation, an assumption we made was that absence of symptoms was a suitable basis for investigating the validity of Root et al. [1, 2]. It is implicit that in the Root et al. model the normal foot is the basis for being symptom free, but normal was largely defined in mechanical terms, with a normal foot demonstrating specific preferred mechanical alignments and movements. However, the basis for the model was its validity in clinical practice and the purpose of practice is prevention or management of symptoms. Clinical practice is not about realignment of structures that are symptom free unless there is compelling evidence that future symptoms are very likely to occur and the consequences of not acting now are significant, and no such evidence exists. Therefore, we assumed absence of symptoms was the best and most externally valid definition of “normal” as a basis for practice.

## Conclusion

None of the static examinations advocated by Root et al. [1, 2] and investigated in this study led to identification of foot deformities that were related to altered foot kinematics. These examinations are routinely used in clinical practice, but the results from this study and allied literature provide little support for their continued use. As such, we believe the Root et al. [1, 2] description of foot function and the associated assessment protocol are not a sound basis for clinical evaluation of the foot nor orthotic prescription.

## Abbreviations

ANOVA: Analysis of variance
BMI: Body mass index
FPI: Foot posture index
LCS: Local co-ordinate system
MPJ: Metatarsophalangeal joint
NCSP: Neutral calcaneal stance position
RCSP: Relaxed calcaneal stance position
